# Epidemiological Characteristics of *Helicobacter pylori* Infection in Children in Northeast Romania

**DOI:** 10.3390/diagnostics13030408

**Published:** 2023-01-23

**Authors:** Ancuta Lupu, Ingrith Crenguta Miron, Andrei Tudor Cernomaz, Cristina Gavrilovici, Vasile Valeriu Lupu, Iuliana Magdalena Starcea, Anca Lavinia Cianga, Bogdan Stana, Elena Tarca, Silvia Fotea

**Affiliations:** 1 Pediatrics, “Grigore T. Popa” University of Medicine and Pharmacy, 700115 Iasi, Romania; 2 3rd Medical Department, “Grigore T. Popa” University of Medicine and Pharmacy, 700115 Iasi, Romania; 3 Pediatric Surgery, “Grigore T. Popa” University of Medicine and Pharmacy, 700115 Iasi, Romania; 4 Medical Department, Faculty of Medicine and Pharmacy, “Dunarea de Jos” University of Galati, 800008 Galati, Romania

**Keywords:** gastritis, *H. pylori*, child, endoscopy, epidemiology

## Abstract

(1) Background: Although gastritis has been associated with multiple etiologies, in pediatrics the main etiology is idiopathic. Many studies have reported mild-to-severe gastritis *Helicobacter pylori* (*H. pylori*) as an etiological factor. We evaluated the distribution of the infection with *H. pylori* by age, gender and place of living; (2) Methods: A retrospective study was conducted over a period of 3 years, over a cohort of 1757 patients of both sexes, aged between 1 and 18 years, admitted to a regional gastroenterology center in Iasi, Romania, with clinical signs of gastritis which underwent upper gastrointestinal endoscopy. The research was based on the analysis of data from patient observation charts and hospital discharge tickets, as well as endoscopy result registers; (3) Results: Out of the 1757 children, in 30.8% of cases the *H. pylori* infection was present. Out of them, 26.8% were males and 73.2% females. The average age of children with an *H. pylori* infection was higher (14.1 + 2.8 DS), compared with children without *H. pylori* (12.8 + 3.7 SD), an average difference of 1.3 years (95% confidence interval 0.96 to 1.66; *p* < 0.001). By place of living, children with *H. pylori* infection were from urban areas at 24.7% and from rural areas at 75.3%; (4) Conclusions: *H. pylori* infection incidence is still high in children, especially in teenagers, so extensive prevention and treatment programs are needed.

## 1. Introduction

Acute gastritis is a term that covers a wide spectrum of entities that induce inflammatory changes in the gastric mucosa. Some etiologies share the same general clinical presentation, but they differ histologically [1]. Some conditions (*Helicobacter pylori (H. pylori)*, inflammatory bowel disease, allergic gastroenteritis) that injure the gastric mucosa can lead to inflammation. Thus, gastritis, as suggested by the suffix -itis, is characterized by the presence of inflammatory cells [2]. The inflammation of the gastric and/or duodenal mucosa is the end result of an imbalance between defensive and aggressive mucosal factors. The degree of inflammation and the presence of this imbalance may subsequently result in varying degrees of gastritis and/or ulceration of the mucosa [3].

Although gastritis has been associated with multiple etiologies, in pediatrics the main etiology is idiopathic. Many studies have reported mild-to-severe gastritis *H. pylori* as an etiological factor. [4].

*H. pylori* is a Gram-negative microaerophilic bacterium which colonizes the gastric mucosa generally in childhood and can determine chronic active gastritis, peptic ulcer disease, gastric cancer and mucosa-associated lymphoid tissue lymphoma later on during adulthood [5]. Its transmission route is still partially unclear, but the infections occur as a result of direct human-to-human transmission or environmental contamination [6]. The increased number of siblings, the education level of the parents, the water sources and garbage collection are also known to be representing important risk factors for the *H. pylori* infection among the pediatric population [7].

It is known that the rate of infection with *H. pylori* reaches a percentage of 50% of the total population and approximately one-third of all children around the world with a high prevalence in low-income countries and in the absence of sanitary conditions, the incidence of infection being severely influenced by the socioeconomic status [8]. In their review, Zabala et al. made an examination of the data from seven cohort studies and showed that the rate of the infection with *H. pylori* in healthy children under 5 years of age remained between 20% and 40% in high-income countries, whereas in the upper-middle income ones, the infection rates variated between 30% and 50%. These data suggest the importance of the country of birth concerning the prevalence of the infection [9]. Additionally, Venneman et al. found in their complex review that in Europe the *H. pylori* infection reached its highest rates in Eastern and Southern Europe which represent, as well, the regions with the highest stomach cancer incidence rates in the European Union [10]. Evidently, the clinical outcome of an *H. pylori* infection depends on multiple favorizing circumstances such as the virulence factors or the host gastric mucosal factors [11].

Clearly, the importance of an early diagnostic of the *H. pylori* infection is undeniable as it can prevent complications in adulthood and implicitly the apparition of gastric cancer [12].

In children, the guidelines recommend that the diagnosis of *H. pylori* infection be based on positive culture or gastritis with *H. pylori* on histopathology with at least one other positive test based on biopsy [13]. The authors of a recent study from Iraq regarding detection of *H. pylori* infection by invasive and non-invasive techniques in adults concluded markedly the role of real-time PCR as more sensitive and accurate than other diagnostic methods because it offers several advantages over culture [14].

Once diagnosed, an *H. pylori* infection has to be eradicated, but the efficacy of the regimen consisting of a standard triple therapy involving antibiotics as amoxicillin, clarithromycin and metronidazole along with a proton pump inhibitor seems to be decreasing lately due to *H. pylori*-resistant strains [15]. Thus, eradicating *H. pylori* infection is starting to become a challenge for all the pediatricians all around the world [16].

As for the epidemiological aspects of the treatment, the results of the EuroPed*HP* Registry 2013 to 2016 showed that the primary antibiotic resistance rates may vary significantly across the geographical regions and that they can also be correlated with the migrant status [17]. In the present study, we aimed to evaluate the cases’ distribution based on sex, age and environmental sources of the *H. pylori* infection. Although its prevalence seems to decrease lately, *H. pylori* infection remains an important public health problem in Romania, and epidemiological studies on its impact among the pediatric population in our country are limited. We evaluated the data from a certain region of Romania, namely the northeast of the country, trying to identify the particularities of *H. pylori* infection among children in relation to their age, gender and environment of origin.

## 2. Materials and Methods

A retrospective study was carried out over a period of 3 years on a cohort of 1757 patients of both sexes, aged between 1 and 18 years, mainly hospitalized in the Gastroenterology Pediatric Clinic, but also in the other clinics of the Emergency Hospital for Children “St. Maria” in Iasi, Romania, with symptoms suggestive of gastritis or gastroduodenal ulcer such as upper abdominal pain, abdominal distension, dyspepsia, nausea or vomiting, in which superior digestive endoscopy (SDE) was performed. Some of them had a positive fecal *H. pylori* antigen in ambulatory. Our hospital is the only one in the northeast of Romania where SDEs can be performed, so our results reflect the epidemiological situation of the pediatric population suffering from this disease in this area with great accuracy.

The main criterion for inclusion in the study was the definite diagnosis of the disease by performing SDE with biopsies taken from the gastric and/or duodenal mucosa. Intravenous sedation was given and standard upper gastrointestinal endoscopy, using the Olympus and Pentax video pediatric gastro-duodenoscopes, was performed to identify the macroscopic changes. General anesthesia in children aged below 10 years of age was used. We obtained 2 biopsies from the antrum, 2 biopsies from the corpus for the histopathological evaluation and 1 biopsy from antrum for the rapid urease test [18,19]. The gastritis was graded according to Houston-updated Sydney system: absent inflammation (Grade 0), mild inflammation (Grade 1), moderate inflammation (Grade 2) and severe inflammation (Grade 3).

During this period, 2042 SDEs were conducted, out of which we excluded 256 SDEs that were performed for verifying the response to therapy rather than for initial diagnosis purposes. Out of the 1786 children for which SDE was performed for diagnosis, we excluded another 29 children who did not have complete data in the observation files. The study was conducted on a final number of 1757 patients.

The research was based on the analysis of data from hospital discharge tickets, patient observation charts and endoscopy result registers. The data regarding the batch considered for the study were organized into a table structure containing a number of 90 category variables and 2 continuous variables. The processing of these data was performed using the SPSS 17.0 platform as well as Excel 2016 software. Chi Squared was calculated to asses association of independent values. Cox regression models adjusting for patient age, sex and area of origin were used characterize the relationship in pediatric patients with gastritis and the probability of testing HP-positive.

All patient’s caregivers have given written informed consent and the “St. Mary” Children Emergency Hospital Ethics Committee’s approval was obtained for publishing this study (31490/29 October 2021).

## 3. Results

We evaluated 1757 patients, out of which 1210 were females and 547 males, whereas 1114 of them originated from rural areas and 643 patients were from urban areas.

Of the 1757 children diagnosed in our study with various forms of gastritis and/or gastroduodenal ulcers, 542 of them (30.8%) had an associated *H. pylori* infection, while the other 1215 (69.2%) did not have the infection at the time of diagnosis. All 542 children were confirmed with *H. pylori* infection by endoscopy with biopsies, all of whom underwent rapid urease testing (RUT).

**Figure 1:**
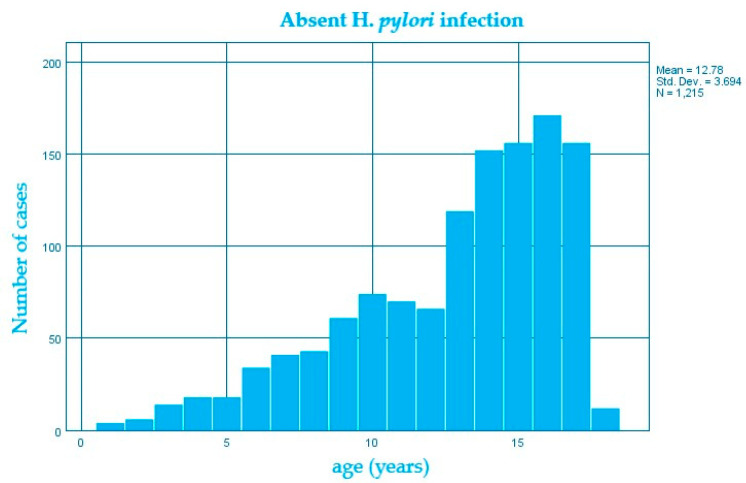
Structure of the group of patients without *H. pylori* infection by age (years).

**Figure 2:**
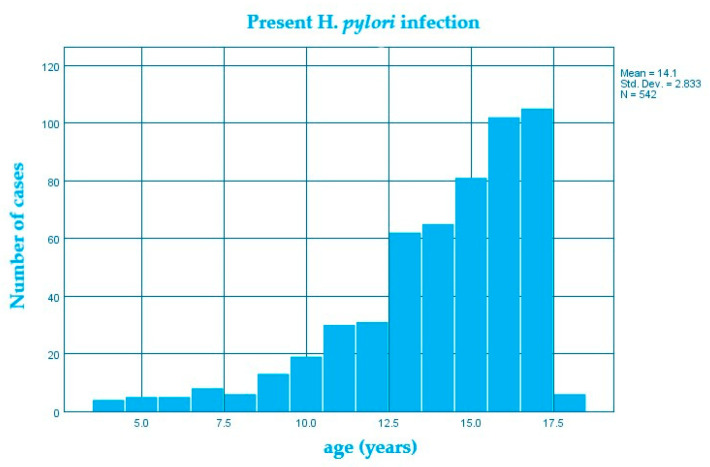
Structure of the group of patients with *H. pylori* infection by age (years).

In the studied group, the average age of children who had an *H. pylori* infection was higher (14.1 ± 2.8 DS) than in those without an *H. pylori* infection (12.8 ± 3.7 DS) (Figure 1 and Figure 2), the average difference being 1.3 years; confidence interval 95% 0.96–1.66; *p* < 0.0001 (Table 1).

**Figure 3:**
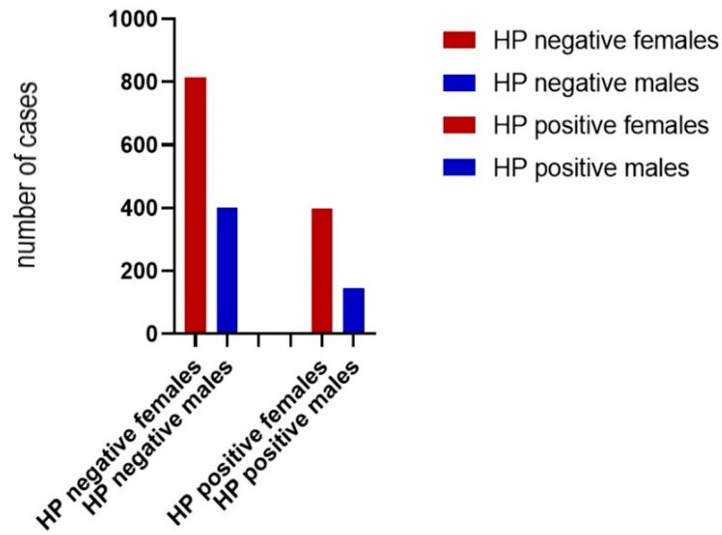
Structure of the batch of patients with or without *H. pylori* infection by gender.

In the studied batch, the female gender represented approximately two-thirds of the entire batch with a percentage of 68.9% (1210 female children), compared to one-third represented by the male gender with a percentage of 31.1% (547 children). The distribution of children with an *H. pylori* infection according to the gender variable revealed a frequency of 26.6% for boys and a frequency of 32.8% for girls (Figure 3). From the statistical analysis, it was concluded that there was a significant difference of this association (χ^2^, *p* = 0.009) (Table 2). The possibility for females displaying gastritis with *H. pylori* is 1.34 times higher as compared to males (OR = 1.34).

The first column represents females with *H. pylori* infection (32.81%) and without *H. pylori* infection (67.19%). The second column describes males with HP infection (26.51%) and without HP infection (73.49%). Chi Squared test of independence was performed and showed there was significant association between gender and *H. pylori* infection (χ^2^ = 7.01, *p* = 0.008).

**Figure 4:**
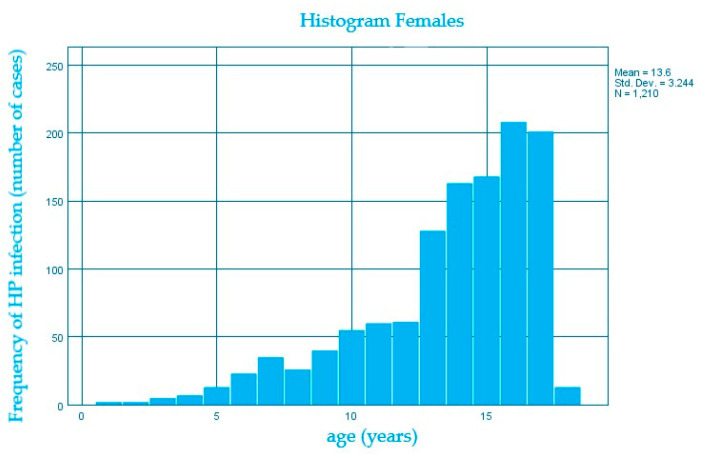
Distribution of *H. pylori* infection (number of cases) according to age (years) in females.

**Figure 5:**
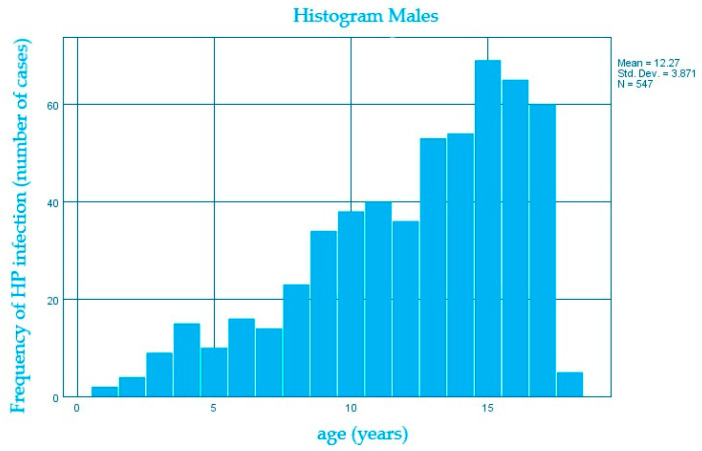
Distribution of *H. pylori* infection (number of cases) according to age (years) in males.

We also evaluated the distribution of the *H. pylori* infection according to age within both genders and we noticed that the number of cases increases along with the median age of 13.6 years for females compared to 12.2 years for males, the average difference being 1.4 years. (Figure 4 and Figure 5)

**Figure 6:**
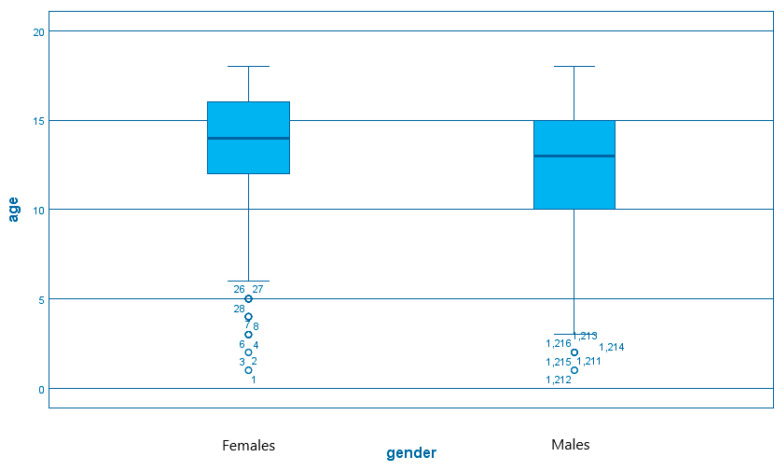
The distribution of age (years) within gender variables in *H. pylori* infection.

A direct comparison between the median age of debut of the *Helicobacter pylori* infection for males and females demonstrates similarities between both genders (Figure 6).

According to patients’ backgrounds, out of the total number of 1757 patients, we observed that 1114 of them (63.4%) originated from the rural areas, whereas 643 of them originated from urban areas with a percentage of 36.6%. (Table 3). From the statistical analysis, it was concluded that there was a powerfully significant difference of this association (χ^2^; *p* < 0.0001) (Table 3). The presence of gastritis with *H. pylori* in patients from rural environments is 2.2 times higher than in patients from urban environments (OR = 2.2).

The first column represents patients originating from rural environment with *H. pylori* infection (36.6%) and without *H. pylori* infection (63.4%). The second column describes children originating from the urban areas with HP infection (20.8%) and without HP infection (79.2%). Chi Squared test of independence was performed and showed there was significant association between environment of origin and *H. pylori* infection (χ^2^ = 47.62, *p* = 0.0001).

**Figure 7:**
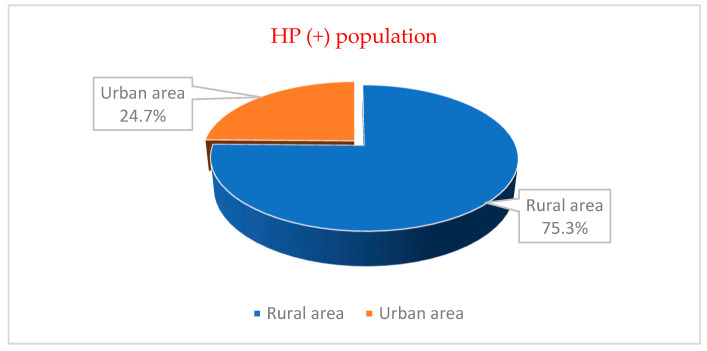
The structure of the group of patients according to the place of living for *H. pylori* present.

The distribution of children with an *H. pylori* infection according to the origin variable showed a frequency of 24.7% in the urban environment and a frequency of 75.3% in the rural environment (Figure 7).

Logistic regression was used to characterize the relationship between age, gender and living conditions (stratified as urban/rural area) for pediatric gastritis patients and the probability of testing *H. pylori*-positive. The most parsimonious model included only age; the results from the model indicate an increased risk of *H. pylori* infection mirroring the aging process. A more complex model included age and living conditions with a similar log likelihood ratio suggesting an increased risk of *H. pylori* infection associated with rural living conditions—odds ratio ~2. A summary of the logistic regression results is shown in Table 4. Adding gender to a third model was considered but the result was not statistically significant (*p* = 0.34) (Wald).

## 4. Discussion

The results of our analysis confirm that almost one-third of the children enrolled in the study have a *H. pylori* infection but unfortunately, in the Romanian medical literature the data regarding this subject are not updated and comparisons are difficult to be made within our country’s territory. However, at the national level, the prevalence of *H. pylori* infection seems to be decreasing lately.

Usually, an *H. pylori* infection is acquired during childhood and persists as chronic gastritis if the organism is not eradicated. With the progress of gastritis over the years, the gastric mucosa undergoes a series of changes that can lead to glandular atrophy, intestinal metaplasia and with increased risk of gastric dysplasia and carcinoma [1,20,21]. On the other hand, a recent study conducted in China identified an inverse relationship between *H. pylori* and asthma, indicating that this infection may represent a protective factor for asthma (OR 1.887–2.008, *p* < 0.05) [22]. This affirmation is supported by the results of a meta-analysis by Chen et al. who observed the same inverse association between the CagA(+) strains of *H. pylori* and the risk of childhood asthma (OR = 0.58; CI, 0.35–0.96, *p* = 0.034) [23].

In the United States, the prevalence of gastritis with *H. pylori* in children appears to be age-dependent. Below the age of 5, few cases are reported but prevalence increases with age, becoming the most common cause of gastritis in adolescents [1,2,24].

The authors of a recent systematic review and meta-analysis regarding global prevalence of pediatric *H. pylori* infection reported that this was present in 32.3% of children and it was higher in low-income and middle-income countries than in high-income countries (43.2% vs. 21.7%). Additionally, the prevalence of infection was higher in older children than in younger ones (41.6% in 13–18-year-olds; 33.9% in 7–12-year-olds; 26.0% in 0–6-year-olds). *H. pylori* infection in children was associated with lower economic status, more children, room sharing, no access to a sewage system, having parents infected with *H. pylori*, drinking non-treated water and adolescents [25].

It has been suggested that gastric pathology (gastritis and ulcer) has become more prevalent in Western countries in the nineteenth century due to a change in the epidemiology of the *H. pylori* infection [24]. Starting from this hypothesis, from environmental changes (which can cause changes in the gastritis pattern) and from current nutrition, we have studied some aspects of gastritis and ulcers in children.

The gastric pathology associated with *H. pylori* infection was more common among adolescents, with 17-year-olds registering the highest frequency with an average age of 14.1 ± 2.8 DS. This distribution could be explained by adolescents’ diet consisting of fast food, carbonated juices, alcohol and coffee, which can exacerbate the symptoms of gastritis with *H. pylori* [25,26]. Their better compliance with digestive endoscopy with the possibility of clear diagnosis could also contribute to the deviation of the frequency to the adult age. There are studies that reported spontaneous elimination of the infection with age, thus explaining the lower prevalence of infection at age 10 [27]. Some authors also argue that the lower prevalence of infection in adolescence age could be explained by an increased attention to health problems in this age group and the use of antibiotics for other infectious diseases [28,29]. In our case, we cannot support these hypotheses, as the prevalence of infection increased in relation to age. The low prevalence at the age of 18 cannot be taken into account, given the lower number of children we have examined, since at this age most patients with similar symptoms resorted to adult gastroenterology exams.

In our study, the female sex was affected in 68.9% of cases (1210 girls out of a total of 1757) and 32.8% had the bacteria present. This may result from the fact that girls give more importance to the symptoms they have, thus increasing the addressability to the doctor. In contrast with our results, Ibrahim et al. show in their meta-analysis that the *H. pylori* infection was more frequent in males than in females (102 studies, OR = 1.06, 95%CI: 1.01, 1.12, I^2^ = 43.7%) [30]. Other studies did not find statistical differences between females and males [31,32,33].

According to the place of living, our statistical analysis showed that there is a higher prevalence of *H. pylori* infection among the children originating from the rural environments with a percentage of 63.35% in northeast Romania. Our data are in agreement with the results obtained by Melit et al. in their study conducted on 137 patients from Romania where the *H. pylori* infection rate was more important in the rural environment than in the urban areas (*p* = *0*.0089) [34]. However, our results are not entirely consistent with a similar analysis made recently in a center in the northwest part of Romania which proved that there were no statistically significant differences between the prevalence of *H. pylori* infection in the rural areas (42.29%) versus the urban environment (39.75%) (*p* = 0.6) [35].

The higher frequency of infection in the rural environment could be explained by the lower socio-economic level and by the larger families in this environment, which promotes the spread of the bacterium. Several studies have reported a higher prevalence of the infection in large families [36,37].

More than 50% of the world’s population is infected with *H. pylori*, which is almost always acquired in the first 5 years of life [38]. In developed countries, the prevalence varies between 1.2% and 12.2% [39,40]. In developing countries, the prevalence is higher. In Indian children, the prevalence of *H. pylori* infection was reported to be 45% [41]. In Bolivia, seroprevalence at age 9 was 70% and in Alaska 69% [40,41].

In Romania, the authors of a retrospective study conducted in Cluj-Napoca, on 194 children, reported the general prevalence of *H. pylori* infection as 36.6% [42]. In another study in Romania, in Targu Mures, conducted on 1041 children aged from 2 to 18 years, the prevalence was similar, of 33.05% [43].

In our study, we found a prevalence of *H. pylori* infection of 30.85%, similar to that reported in the other Romanian studies. The prevalence of the bacteria was roughly the same over the three years of study.

Initially, the specific guidelines for eradicating *H. pylori* infection were limited to peptic ulcer, but in 1997, the “Digestive Health Initiative” (DHI) during the “International Update Conference on *H. pylori*” extended the recommendations for testing and treating *H. pylori* [44]. Thus, the recommendations for *H. pylori* treatment are as follows: in the presence of *H. pylori*-associated peptic ulcer, treating *H. pylori* infection in the absence of peptic ulcer in children with dyspeptic symptoms may be considered, a “test and treat” strategy is not recommended in children based on non-invasive methods, in children infected with *H. pylori* who have a first-degree relative with gastric cancer, treatment may be recommended and the monitoring of antibiotic resistance rates of *H. pylori* strains in children and adolescents is recommended in different countries and geographical areas [45].

For the diagnosis, we performed SDE with biopsies taken from the gastric and/or duodenal mucosa such as in the recommendation of the new guidelines. The guidelines recommended that the initial diagnosis of *H. pylori* infection should not be based on noninvasive tests. A positive bacterial culture or *H. pylori* gastritis on histopathology with at least one other positive test such as rapid urease test, or molecular-based assays (polymerase chain reaction or fluorescent in situ hybridization) are necessary [13].

*H. pylori* has been shown to be highly resistant to clarithromycin in both children and adults, which is why studies at all ages recommend testing for antimicrobial susceptibility in *H. pylori* using molecular biopsy-based techniques, such as real-time PCR [13,46]. In our center, this test is not available and we considered it a limitation for our study. This is added to the unavailable laboratory investigations due to the fluctuation of existing funds (the study of vacA and cagA strains of *H. pylori*, the analysis of *H. pylori* antigen from fecal samples throughout the study period) and the impossibility of long-term follow-up of patients due to their very large number, but also to the lack of cooperation on their part.

## 5. Conclusions

The infection rate with *H. pylori* is still high in children, especially in teenagers, so extensive prevention and treatment programs are needed. An early diagnosis can significantly minimize complications during adulthood and, undoubtedly, can present an important impact on the socio-economic status in Romania.

## Figures and Tables

**Table 1:** Average age of diagnosis of infection with *H. pylori*.

Age (Years)	Frequency Total	*H. pylori* Negative	*H. pylori* Positive	Pearson Chi Square	Likelihood Ratio	*p* Value
1	4 (0.2%)	4 (100%)	0	61.7	72.33	*p* < 0.0001
2	6 (0.3%)	6 (100%)	0			
3	14 (0.8%)	14 (100%)	0			
4	22 (1.3%)	18 (81.8%)	4 (18.2%)			
5	23 (1.3%)	18 (78.3%)	5 (21.7%)			
6	39 (2.2%)	34 (87.2%)	5 (12.8%)			
7	49 (2.8%)	41 (83.7%)	8 (16.3%)			
8	49 (2.8%)	43 (87.8%)	6 (12.2%)			
9	74 (4.2%)	61 (82.4%)	13 (17.6%)			
10	93 (5.3%)	74 (79.6%)	19 (20.4%)			
11	100 (5.7%)	70 (70%)	30 (30%)			
12	97 (5.5%)	66 (68%)	31 (32%)			
13	181 (10.3%)	119 (65.7%)	62 (34.3%)			
14	217 (12.4%)	152 (70%)	65 (30%)			
15	238 (13.5%)	157 (66%)	81 (34%)			
16	273 (15.5%)	171 (62.6%)	102 (37.4%)			
17	260 (14.8%)	152 (59.6%)	105 (40.4%)			
18	18 (1%)	12 (66.7%)	6 (33.3%)			
Total	1757 (100%)	1215 (69.2%)	542 (30.8%)			

**Table 2:** Estimated parameters in testing the association between *H. pylori* infection and the gender variable.

	Female	Male	Pearson Chi Square	Likelihood Ratio	*p* Value
HP positive	397 (32.81%)	145 (26.51%)	7.01	7.13	*p* = 0.008
HP negative	813 (67.19%)	402 (73.49%)			
Total	1210 (100%)	547 (100%)			

**Table 3:** Estimated parameters in testing the association between *H. pylori* infection and the origin variable.

	**Rural** **(Number/Percentage)**	**Urban** **(Number/Percentage)**	**Pearson Chi Square**	**Likelihood Ratio**	***p*** **Value**
HP positive	408 (36.6%)	134 (20.8%)	47.62	49.38	*p* < 0.0001
HP negative	706 (63.4%)	509 (79.2%)			
Total	1114 (100%)	643 (100%)			

**Table 4:** Logistic regression model including age, living conditions and *H. pylori* infection status for pediatric patient with gastritis.

	B	S.E.	Wald	df	Sig.	OR	95% CI for OR	
							Lower	Upper
Age (years)	0.11	0.02	44.25	1	0.000	1.12	1.08	1.16
Urban area			39.05	1	0.000			
Rural area	0.73	0.12	39.05	1	0.000	2.08	1.65	2.61
Constant	−2.84	.25	126.09	1	0.000	0.06		

B = beta coefficient; S.E. = standard error; df = degrees of freedom; Sig. = statistical significance; OR = odds ratio; CI = confidence interval.

## Data Availability

The data presented in this study are available on request from the corresponding author.
